# Resveratrol Protects against Skin Inflammation through Inhibition of Mast Cell, Sphingosine Kinase-1, Stat3 and NF-κB p65 Signaling Activation in Mice

**DOI:** 10.3390/ijms24076707

**Published:** 2023-04-04

**Authors:** Christopher D. Carlucci, Yvonne Hui, Alena P. Chumanevich, Piper A. Robida, John W. Fuseler, Mathew Sajish, Prakash Nagarkatti, Mitzi Nagarkatti, Carole A. Oskeritzian

**Affiliations:** 1 Department of Pathology, Microbiology and Immunology, University of South Carolina School of Medicine, Columbia, SC 29209, USA; 2 Department of Drug Discovery and Biomedical Sciences, College of Pharmacy, University of South Carolina, Columbia, SC 29208, USA

**Keywords:** mast cells, resveratrol, skin inflammation, quantitative imaging, chemokines, sphingosine kinase 1, Stat3, NF-κBp65

## Abstract

Inflammation is pathogenic to skin diseases, including atopic dermatitis (AD) and eczema. Treatment for AD remains mostly symptomatic with newer but costly options, tainted with adverse side effects. There is an unmet need for safe therapeutic and preventative strategies for AD. Resveratrol (R) is a natural compound known for its anti-inflammatory properties. However, animal and human R studies have yielded contrasting results. Mast cells (MCs) are innate immune skin-resident cells that initiate the development of inflammation and progression to overt disease. R’s effects on MCs are also controversial. Using a human-like mouse model of AD development consisting of a single topical application of antigen ovalbumin (O) for 7 days, we previously established that the activation of MCs by a bioactive sphingolipid metabolite sphingosine-1-phosphate (S1P) initiated substantial skin remodeling compared to controls. Here, we show that daily R application normalized O-mediated epidermal thickening, ameliorated cell infiltration, and inhibited skin MC activation and chemokine expression. We unraveled R’s multiple mechanisms of action, including decreased activation of the S1P-producing enzyme, sphingosine kinase 1 (SphK1), and of transcription factors Signal Transducer and Activator of Transcription 3 (Stat3) and NF-κBp65, involved in chemokine production. Thus, R may be poised for protection against MC-driven pathogenic skin inflammation.

## 1. Introduction

Inflammation constitutes a critical component of skin pathologies and allergic skin responses, such as atopic dermatitis (AD) or eczema [1]. AD is a chronic inflammatory skin disease with complex pathogenesis, featuring severe itch and skin lesions. While treatment options are expanding, they remain costly and may cause severe adverse effects [1]. Thus, there is an unmet medical need to develop safe therapies aiming to prevent the spreading of skin lesions and progression to chronic disease.

Resveratrol (trans-3-4′,5-trihydroxystilbene, R) is an extensively studied natural polyphenolic phytoalexin enriched in red grapes that has gained attention for its overall anti-inflammatory effects [2,3,4,5,6,7]. However, animal studies and human clinical trials have yielded contrasting results with beneficial, lack of beneficial, or even adverse effects on health, owing to differences in experimental models, R bioavailability and pharmacokinetics [8], dosing [9,10], formulation, and circadian variation [2]. Additionally, R exerts contrasted effects depending on the prevalent stereoisomer present in solution [2,9].

Mast cells are skin-resident innate immune cells that initiate many aspects of inflammatory responses due to strategic tissue residence in close proximity to blood vessels and have a unique ability to synthesize and store an arsenal of bioactive substances [11,12,13]. Released upon activation, a process also named degranulation, MC mediators regulate inflammation by recruiting cells from the circulation, as well as acting on neighboring cells to orchestrate tissue remodeling [14,15,16,17,18,19,20]. We have previously reported inflammation-enabling functions of bioactive sphingolipid sphingosine-1-phosphate (S1P)-activated MCs in early-phase AD in mice, suggesting that controlling MC reactivity may prevent the development of AD [16,18]. It has been reported that R could inhibit MC inflammatory functions by reducing the expression of the high-affinity receptor for immunoglobulin (Ig)E, *FcεRI*, on human Laboratory of Allergic Diseases (LAD)2 MC line [21], preventing an increase in MC numbers in allergic enteritis, colitis [22], passive cutaneous anaphylaxis [23], and signal transducer and activator of transcription 3 (Stat3) activation [24]. Intriguingly, R was reported to not only stimulate S1P signaling [25] but also to inhibit S1P-producing sphingosine kinase 1 (SphK1) activity [26]. Moreover, R was shown to differentially impact human MC activation by targeting the arachidonic acid pathway and subsequent prostaglandin D2 synthesis but also by enhancing TNF release [27], whereas others reported an overall reduction in MC activation [28].

In this study, we hypothesized that R may attenuate the early inflammatory skin changes at the inception of AD by maintaining homeostasis and preventing the activation of MCs and signaling pathways. To this end, the inflammation-triggering antigen ovalbumin (O), the most abundant protein in egg white widely used in allergy models because of its strong allergenicity [29], was applied topically on mouse skin in the presence or absence of R, as previously described [16,18], and histopathological, cellular, and molecular evaluation of treated skin samples was conducted to measure inflammatory responses and signaling pathways.

## 2. Results

### 2.1. Morphometric Method of Skin Layer Thickness

**Figure 1:**
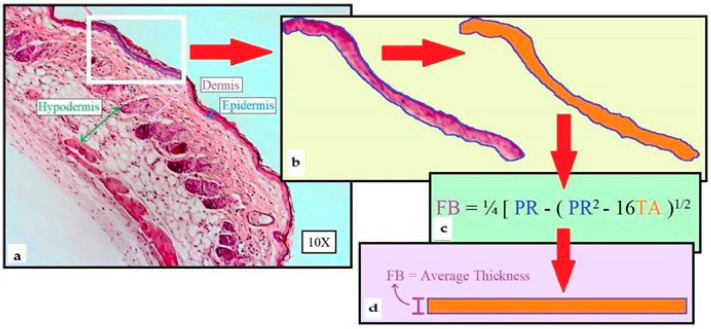
Flow chart for computer-assisted measurements of skin layer thickness: (**a**) H&E-stained whole skin section, showing individual layer thickness: epidermis (blue), dermis (purple), hypodermis (green), and Region of Interest (ROI, white rectangle) used to survey each full-length layer. (**b**) Epidermis isolation within the ROI converted into a Traced Area (TA, orange). (**c**) Equation used to calculate layer thickness (FB, Fiber Breadth) using measured perimeter of the isolated layer (PR, μm) and TA (μm^2^). (**d**) Resulting conversion of the irregular fiber (**b**) into a rectangle measuring the average thickness of the layer while preserving its original length-to-width ratio.

Using our expertise in quantitative imaging, we developed a new computer-aided analysis of H&E-stained whole skin sections mounted on microscopy slides imaged at 10× magnification, allowing the thickness of the different skin layers in variously treated samples to be accurately and objectively measured (see Section 2.2). Our morphometric method was based on isolating each layer (epidermis, dermis, and hypodermis) within a region of interest (ROI, e.g., white rectangular frame in Figure 1a for epidermis thickness measurement), then surveying each full-length layer and collect measurements. Figure 1b shows the isolation of the epidermis within the ROI, converted into an irregular fiber, and depicted in orange by the MetaMorph^®^ 601 Microscopy Automation and Image Analysis software (Molecular Devices, Sunnydale, CA, USA). The insert in Figure 1a was enlarged for clarity. Figure 1c demonstrates the equation employed to calculate the actual layer thickness (Fiber Breadth, FB) through software-generated Traced Area (TA depicted in orange in Figure 1b, expressed in μm^2^) and Perimeter (PR, delineated in blue in Figure 1b, expressed in μm) quantifications, converting the irregular fiber imaged in Figure 1b into a rectangle (Figure 1d) while preserving the original length-to-width ratio (i.e., average thickness data).

### 2.2. Resveratrol Prevents Epidermal Thickening Triggered by Exposure to Ovalbumin

**Figure 2:**
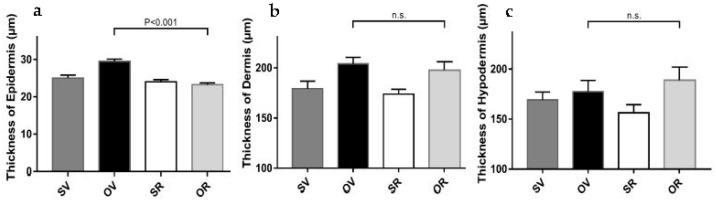
Resveratrol prevents ovalbumin-induced epidermal but not dermal layer thickening: (**a**) Thickness measurements of the epidermis, (**b**) dermis, and (**c**) hypodermis of mouse dorsal skin after 7 days of epicutaneous exposure to saline (S) and vehicle for resveratrol (V) (dark grey filled bars), ovalbumin (O) and V (black filled bars), S with resveratrol (R) (empty bars), or O with R (light grey bars). *N* = 3 mice, 3–6 images per mouse, 3 ROI per image for each experimental group. n.s., not significant.

We previously reported that a single epicutaneous exposure to O for 7 days yielded thickening of the epidermis and the dermis layers in the dorsal skin, compared to saline (S) controls, using a well-established mouse model of AD [16]. In the current study, we applied the method newly described in Section 2.1 (Figure 1) to measure the thickness of each skin layer upon exposure to O in the presence and absence of resveratrol (R) (Figure 2). O supplemented with the ethanol vehicle (V) for resveratrol (R) induced the thickening of the epidermis (Figure 2a) and the dermis (Figure 2b), compared to controls (SV). However, R prevented O-induced epidermal (Figure 2a) without altering dermal thickening (Figure 2b).

The thickness of the hypodermis layer was unaltered upon O exposure, as previously observed [16], including in the presence of R (Figure 2c).

### 2.3. Resveratrol Treatment Precludes Ovalbumin-Induced Cell Infiltration in the Hypodermis

**Figure 3:**
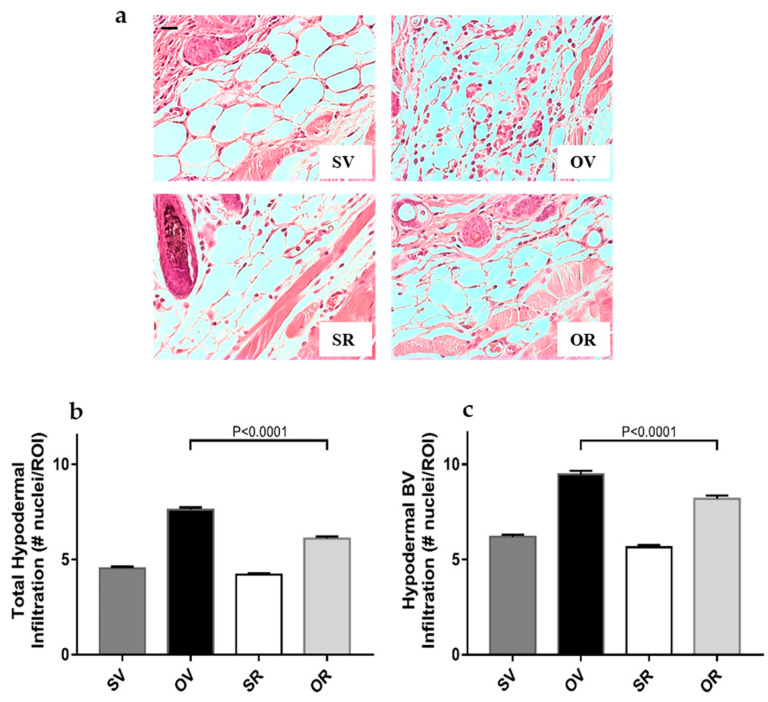
Decreased cellular infiltration in the hypodermis of mouse dorsal skins after a 7-day treatment with ovalbumin and resveratrol: (**a**) Hematoxylin and eosin (H&E) staining of skin tissues, focusing on the hypodermis after 7 days of epicutaneous exposure to saline (S) and vehicle for resveratrol (V), ovalbumin (O) and V, S with resveratrol (R), or O with R (scale bar = 50 mm). (**b**) Quantification of whole hypodermal cell infiltration after 7 days of epicutaneous exposure to S and V (dark grey filled bars), O and V (black filled bars), S with R (empty bars), or O with R (light grey bars). *N* = 6 mice per experimental group, 10 images per mouse, 10 ROI per image. (**c**) Quantification of hypodermal cell infiltration around blood vessels (BV) after 7 days of epicutaneous exposure to S and V (dark grey filled bars), O and V (black filled bars), S with R (empty bars), or O with R (light grey bars). #, number of, *n* = 6 mice, 10 images per mouse, 225–255 BV-containing ROI per experimental group.

We previously reported that one week of exposure to O triggered significant infiltration of cells in the hypodermis, compared to saline controls, in a mouse model of AD, including around blood vessels (BV) [16,17]. We applied the computer-assisted cell quantification method described previously [16,17] to measure cell infiltration in the entire hypodermis, as well as around BV (Figure 3).

Representative images of H&E-stained skin samples focusing on the hypodermis illustrated the ameliorative effects of R on O-induced infiltration (Figure 3a). The integration of nuclei numbers enumerated in each ROI was significantly decreased upon resveratrol treated-, O-exposed mouse skins, compared to O alone in the entire hypodermal area (Figure 3b) or around the hypodermal BV (Figure 3c). However, resveratrol application did not restore cellularity to the level observed in saline-treated skin samples.

### 2.4. Resveratrol Treatment Significantly Reduces Mast Cell Activation and Responsiveness in the Hypodermis of Ovalbumin-Treated Mouse Skins

**Figure 4:**
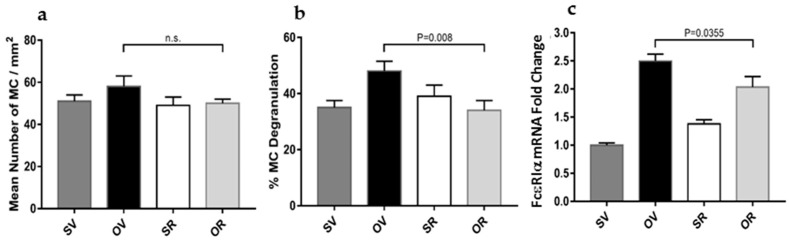
Resveratrol attenuates skin mast cell activation and reactivity without affecting mast cell numbers: (**a**) Mean total mast cell numbers, (**b**) activation (degranulation), and (**c**) *FcεRIα* mRNA expression levels after 7 days of epicutaneous exposure to saline (S) and vehicle for resveratrol (V) (dark grey filled bars), ovalbumin (O) and V (black filled bars), S with resveratrol (R) (empty bars), or O with R (light grey bars). *N* = 3–9 mice per experimental group and 15 images per mouse (**a**,**b**), n.s., not significant.

We previously established a new morphometric imaging method to quantify mammal mast cells (MCs) and MC activation status in microscopy slides [16,17]. In situ quantifications were conducted in differentially treated mouse skins using our computer-aided method to identify MCs (Figure 4a) and distinguish resting from activated (degranulated) MCs (Figure 4b). These results showed that R or its vehicle did not alter the average number of skin MCs per mm^2^, as previously reported. Whereas O treatment did not significantly increase tissue MC numbers (Figure 4a), it significantly augmented MC activation (Figure 4b).

Notably, O treatment significantly increased *FcεRIα* mRNA levels, the sub-unit of the receptor binding IgE with high affinity that is mostly expressed on mast cells in the skin, suggesting a potential functional priming for enhanced reactivity (Figure 4c). Figure 4b,c demonstrated that R normalized MC activation status to a steady state as well as significantly decreased *FcεRIα* mRNA expression.

### 2.5. Resveratrol Treatment Averts Chemokine mRNA Expression Level Increase in the Skin of Ovalbumin-Treated Mouse Skins

**Figure 5:**
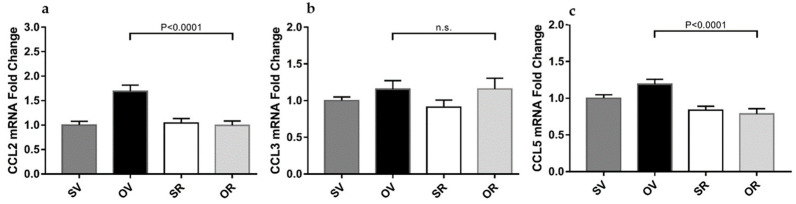
Resveratrol alleviates ovalbumin-induced skin chemokine mRNA elevation without altering *SphK1* mRNA levels: (**a**) skin *CCL2*, (**b**) *CCL3*, and (**c**) *CCL5* mRNA expression levels after 7 days of epicutaneous exposure to saline (S) and vehicle for resveratrol (V) (dark grey filled bars), ovalbumin (O) and V (black filled bars), S with resveratrol (R) (empty bars), or O with R (light grey bars). *N* = 6 mice per experimental group and two independent trials, n.s., not significant.

Previously published studies demonstrated that one-week exposure to O induces increased chemokines *CCL2*, *CCL3*, and *CCL5* skin mRNA levels in female [16] but not in male mice [17]. In the current study, mRNA expression levels featured a similar elevation upon O application and were normalized in skin samples exposed to O and resveratrol for 7 days (Figure 5a,c), except for *CCL3* chemokine (Figure 5b).

### 2.6. Resveratrol Treatment Mitigates Ovalbumin-Induced Skin Inflammation through Inhibition of Sphingosine Kinase 1, Stat3, and NFκB/p65 Activation

**Figure 6:**
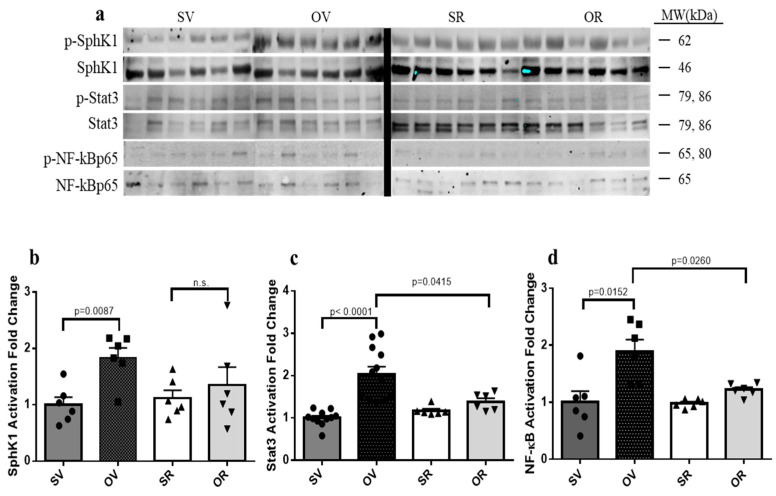
Resveratrol suppresses ovalbumin-induced activation of SphK1, Stat3, and NF-κB: (**a**,**b**) skin SphK1, (**a**,**c**) Stat3, and (**a**,**d**) NF-κB/p65 activation levels after 7 days of epicutaneous exposure to saline (S) and vehicle for resveratrol (V) (dark grey filled bars), ovalbumin (O) and V (black filled bars), S with resveratrol (R) (empty bars), or O with R (light grey bars). *N* = 6 mice per experimental group, n.s., not significant.

We and others previously identified the importance of MC activation by S1P in vivo in mouse models of anaphylaxis [14,15,30,31] and eczema [16]. Importantly, S1P-mediated MC activation was linked to Stat3 activation resulting in cell-recruiting *CCL2*, *CCL3*, and *CCL5* chemokine release [15,16]. Of note, O treatment significantly increased the phosphorylation of SphK1, a mark of enzymatic activation, which was normalized upon exposure to R (Figure 6a,b).

Similarly, Stat3 activation/phosphorylation that was significantly augmented upon O application was reduced to control levels in R-treated skin samples (Figure 6a,c). The transcription factor NF-κB is another critical regulator of inflammatory responses, and its canonical activation occurs through its p65 sub-unit phosphorylation and translocation to the nucleus to exert transcriptional activity on inflammatory genes, including chemokines [32]. Our results substantiated that O-induced activation/phosphorylation of p65 was significantly abated in the presence of R (Figure 6a,d).

## 3. Discussion

We show that resveratrol potently inhibits multiple features of the patho-mechanistic events occurring at the onset of AD. Thus, epidermal thickening that was observed in early-phase AD was prevented by R treatment in a well-established human-like mouse model utilizing the epicutaneous application of antigen O on the dorsal skin [16,17,33,34]. Importantly, we used our established expertise in quantitative imaging [16,17,18,35] to develop a new computer-assisted method extensively described herein, to measure skin layer thickness. Other early inflammatory features, including hypodermal infiltration and MC activation but not local MC numbers, were substantially mitigated upon R treatment. We have previously established that early infiltration of cells in inflamed lungs and skin resulted from the MC-dependent production of *CCL2*, *CCL3*, and *CCL5* chemokines [14,15,16,17,36,37,38,39,40,41,42,43]. In the current study, attenuated O-induced cell infiltration by R was accompanied by the normalization of skin mRNA expression levels of chemokines. Mechanistically, we had previously reported that MC-derived chemokine production was linked to the activation/phosphorylation of Stat3. Ovalbumin-induced Stat3 activation was significantly decreased upon O and R application on mouse skins. In support of this, recent studies established the inhibition of phosphorylation of Stat3 by R in human MCs, also diminishing MC activation and chemokine production [24]. Composed of multiple sub-units that include p65, the transcription factor NF-κB is another critical regulator of inflammatory responses implicated in the upregulated transcription of inflammatory gene-encoding chemokines [32]. We found that R treatment counteracted O-induced p65 activation/phosphorylation, thus strongly suggesting that the ameliorative effects of R may also involve the dampening of NF-κB signaling. In agreement, blocking the transcriptional activity of NF-κB through decreased activation/phosphorylation of the p65 sub-unit by R has been previously reported [44,45]. Finally, sphingosine kinase 1 (SphK1), the most prevalent S1P-producing enzyme, is a known signaling target of R [4,20,46,47]. Our results demonstrate that R precluded activation of SphK1. Supporting the relevance of these results, we have previously established that the early skin changes associated with the onset of AD were negated in SphK1 knockout mice [16]. Perhaps also relevant to the current study, R has been identified as inhibiting SphK1-mediated NF-κB activation [48] and interfering with SphK1 activation through the prevention of its translocation to the plasma membrane [47]. Indeed, SphK1 is prevalently cytosolic [49] and, upon activation/phosphorylation, migrates to the plasma membrane to generate S1P from its substrate sphingosine [50]. We and others have demonstrated that S1P regulates MC responses in multiple ways: differentiation, proliferation, and protease repertoire [38,51] and activation [14,15,16,17,36,37,38,39,40,41,42,43]. Thus, R may thwart MC activation by preventing the activation of SphK1 in this mouse model of AD. Furthermore, our results support the poly-mechanistically beneficial effects of R in developing AD, shedding light on the multiple inflammatory pathways that are impacted and ameliorated through skin treatment with R.

We and others previously established the importance of lipopolysaccharide (LPS) and its receptor Toll-like receptor 4 (TLR4) signaling in the initiation of allergic skin inflammation, strongly suggesting that the early inflammatory steps preceding a diseased state are independent of the antigen [16,17,52]. Notably, R was shown to prevent TLR4 activation [53] and expression [45]. Of note, TLR4 downstream signaling encompasses NF-κB and, similar to Gong et al. [26], we observed a significant inhibition of NF-κB p65 activation as well as noted a significant decrease in Stat3 phosphorylation; an event that we had previously linked to chemokine production in MCs, downstream of MC S1P receptor 2 (S1PR2) [15]. In agreement, Gong et al. reported the inhibition of LPS-induced inflammation by R through the SphK1/S1PR2/NF-κB pathway [26].

This study presents several limitations: We and others have previously reported that AD has a more severe presentation and is more prevalent in female than male mice and human adults [17,54,55,56,57,58,59,60,61,62,63,64]. Therefore, only female mice were utilized in our study. Reactive oxygen species (ROS) also act as signaling molecules in inflammatory disorders [65]. We have not investigated their functions in our model, nor the effects of R on ROS levels. R metabolites were discounted; although, it is known that R rapidly metabolizes in vivo in the liver [66,67,68]. To circumvent this, we opted to apply R daily without disturbing the gauze patch from the treated area of mouse dorsal skin. The R concentration of 2.5 μg/mL was selected based on previous studies [2,9]. This concentration of R is considered moderate and physiologically relevant for in vivo studies as well [69]. A recent study uncovered differences in the inhibitory effects of R in mouse bone marrow-derived MCs and the human Laboratory of Allergic Diseases (LAD)2 MC line [21]. Studies are currently ongoing in our lab aiming to compare the effects of R in mouse and human primary MCs.

Given the broad spectrum of mediators they produce, MCs are recognized as key players, not only in the maintenance of homeostasis [19,20] but also in the initiation of many pathological conditions beyond allergy and anaphylaxis, asthma, and AD, thus also including autoimmunity and cancer onsets [38,61,62,63,64,65,66,67,68,69,70,71,72]. Our results strongly support local MC stabilization, accompanied by decreased levels of chemokines and infiltration by R in otherwise inflamed skin. Therefore, the identification of R as a natural modulator of MCs may pave the way for the development of novel prophylactic strategies for the development of inflammatory and carcinogenic processes.

## 4. Materials and Methods

### 4.1. Atopic Dermatitis Model

AD was initiated in 8 to 12 weeks of age female C57Bl/6J mice exactly as previously described [16,17,18,33]. Mice were purchased from The Jackson Laboratory (Bar Harbor, ME, USA). Mice were randomly assigned as previously described [16,17] to either experimental groups, saline (S)/R vehicle (V), ovalbumin (O)/V; S/R or OVA/R. In the AD model, 100 μL of OVA solution (100 μg OVA (Millipore Sigma, Burlington, MA, USA) in V (0.05% ethanol in saline) (OV group), SV (0.05% ethanol), saline/R (SR, i.e., 2.5 μg R (TCI America/Fisher Scientific, Waltham, MA, USA) in 0.05% ethanol), or OVA/R (OR group, i.e., 100 μg OVA and 2.5 μg R in saline with 0.05% ethanol) was pipetted as a final volume of 100 μL on 1 cm × 1 cm gauze pads (patches) that were applied on the shaved and tape-stripped upper-back areas of mice. A half cm flexible tube (plastic hematocrit capillary tubes, Fisher Scientific, Pittsburgh, PA, USA) was then placed over the patch, with one end over the center of the patch and the other extending slightly past its caudal border. Next, the tube and patch were secured in place with a Tegaderm^®^ transparent dressing (3M HealthCare, St. Paul, MN, USA) covering the patch, ensuring that the caudal end of tubing was flush with the edge of the dressing. An adhesive bandage was then applied over the dressing to keep the patch, dressing, and tube firmly positioned for 7 days [16,17]. Next, mice received the same treatments as above in a final volume of 100 μL onto the patch (i.e., either saline with 0.05% ethanol for the SV and OV groups or 2.5 μg R in saline with 0.05% ethanol for the SR and OR groups) without O daily via a 1 mL syringe and needle inserted into the capillary tube so as not to move the patch from the treated area. The patches were removed after 7 days, and skin samples were harvested from euthanized mice. All animal procedures were performed in accordance with the University of South Carolina Institutional Animal Care and Use Committee (IACUC) approval (IACUC Protocol Number: 2403-101303-010818 approved on 1 August 2018), and all methods used adhered to relevant guidelines and regulations.

### 4.2. R Preparation

R was dissolved in 100% ethanol to its maximum soluble concentration of 50 mg/mL as well as maximum stability [73]. This stock solution was further diluted in 0.9% saline (S) to a concentration of 2.5 mg/mL of S containing 5% ethanol. The final working solution of R of 2.5 μg/mL, a physiologically relevant concentration [9,74], was obtained by additional dilution using either 0.9% S or combined with OVA 100 μg/mL in 0.9% S. Vehicle control groups with 5% ethanol were further diluted in 0.9% S or combined with OVA, as above. Ethanol was used as V for R as physiologically relevant concentrations of R are not attainable in S because of R’s insolubility in nonorganic solvents [69].

### 4.3. Histology, Microscopy, and Morphometric Measurements

Skin samples were fixed in freshly prepared 4% paraformaldehyde, paraffin-embedded, sectioned, and mounted on microscopy slides. Image analyses and quantifications were performed in a single-blinded manner. One slide of each skin sample was stained with hematoxylin and eosin (H&E) for measurement of skin layer thickness and cell infiltration.

Tagged image file format (TIFF) images of the H&E slides were taken with a Nikon E-600 microscope, a Micropublisher camera, and software at 10× magnification. Thickness was measured using the MetaMorph^®^ 6.1 Microscopy Automation and Image Analysis software (Molecular Devices, Sunnyvale, CA, USA) and a morphometric imaging technique recently developed by our lab [35], as explained in Figure 1. Three regions of interest (ROI) were randomly selected for each skin layer per image (3–6 images per animal) [16,17,35]. The MetaMorph software was used to trace the perimeter of each skin layer in each ROI. Next, the software provided measurements for the traced area (TA) and its perimeter (PR). The TA was defined as an irregular two-dimensional fiber and the equation below was used to calculate the average thickness or breadth of each fiber [75].

Fiber Breadth (FB) = ¼ [PR − (PR^2^ − 16TA)^1/2^]

FB measurements were collected to calculate the average thickness of the epidermis, dermis, and hypodermis for each harvested skin sample.

The computer-assisted quantification of cell infiltration was conducted exactly as previously described [16,17,35]. Briefly, a minimum of 10 images per mouse were collected from H&E-stained microscopy slides and analyzed with the MetaMorph 6.1. software. The distinct purple/blue color of hematoxylin-stained nuclei in skin sections was defined using the hue, saturation, and intensity (HSI) color model through a thresholding process [35]. Analyses were performed only of areas satisfying thresholding to eliminate background and nonspecific staining. Nuclei were defined using previously validated morphometric parameters uniquely identifying size and shape restricted to nuclei [16,17,35]. The hypodermis layer was surveyed using a circular ROI of fixed diameter (75 μm) imaging the entire layer, as our previous studies revealed that early cell infiltration occurred in this innermost and most vascularized layer of the skin [16,17,35]. Next, the software quantified the number of nuclei present in each ROI, and the average number of nuclei per ROI was calculated for each experimental cohort [16,17,35].

Local MC activation/degranulation was quantified using previously validated morphometric parameters associated with MCs, which describe MC cytoplasm and distinguish resting from activated MCs [16,17,35]. To this end, 15 adjacent images of the dermis and hypodermis at 40× magnification were recorded as TIFF files from each methylene blue (MB)-stained microscopy slide, a staining that not only specifically identifies MCs [16,17,76] but also their activation status [16,17]. Next, each MC was identified as an individual ROI using the HSI color model thresholding routine of the MetaMorph 6.1 software [35]. HSI values were set to isolate the distinct blue/purple color of MC cytoplasm stained with MB [16,17,76]. The integrated morphometry routine of the MetaMorph 6.1 software was employed to measure the area (A) and the integrated optical density (IOD) of each thresholded ROI [16,17,76]. IOD values represent the total amount of material present in the ROI (i.e., the MCs). The IOD of each ROI was then divided by its area and the resulting calculated values were used to define resting and activated MCs, as previously reported [16,17,35]. Next, the average number of total, resting/intact, and activated/degranulated MCs was calculated, exactly as previously described [16,17,35].

### 4.4. RNA Preparation and Quantitative Reverse Transcription Polymerase Chain Reaction Assay

One strip of skin was snap-frozen in liquid nitrogen immediately after harvesting and stored at −80 °C until use. Total RNA was isolated and purified from each sample with the miRNeasy kit (Qiagen, Valencia, CA, USA), following the manufacturer’s instructions. The cDNA synthesis kit (Bioline/Meridian Bioscience, Memphis, TN, USA) was used according to the manufacturer’s protocol to reverse transcribe cDNA from the isolated RNA. qRTPCR was conducted using the SensiFAST™ SYBR No-ROX kit (Bioline) and the CFX Connect instrument (Bio-Rad, Hercules, CA, USA). Samples were analyzed for the presence of mRNA coding for chemokines *CCL2*, *CCL3*, and *CCL5*, *FcεRIα* and *SphK1*, normalized to the *β2-microglobulin* (*β2M*) and *β-actin* (*βA*) reference genes. Primer sequences are listed in Table 1 (Thermo Fisher Scientific, Waltham, MA, USA).

The qRTPCR conditions were as follows: an initial step at 95 °C for 5 min and each of the 40 cycles consisting of 10 s at 95 °C then 1 min annealing at 55 °C followed by an extension at 72 °C. Each sample was assayed in duplicate determinations. Data analysis was performed with the CFX Manager™ software with normalization to control saline/R vehicle-treated samples, which was directly proportional to the amount of mRNA of the gene of interest relative to the amount of mRNA of the reference genes, i.e., *β2-microglobulin* and *β-actin*.

### 4.5. Western Blot Analysis

Proteins of skin samples were extracted from snap-frozen tissues, and protein concentrations were measured using a Pierce™ BCA Protein Assay Kit (Thermo Fisher). Equal amounts of proteins were electrophoretically separated on 4–20% Mini-PROTEAN^®^ TGX™ gels (Bio-Rad, Hercules, CA, USA) and then transferred to nitrocellulose membranes. Membranes were immunoblotted with primary Ab against phosphorylated (P-) SphK1 (Exalpha X1849P), SphK1 (Bioss bs-2652R), P-Stat3 (Santa Cruz SC-81523), Stat3 (Cell Signaling Technology, Danvers, MA, USA, 4904S), P-p65 (Cell Signaling Technology 3033S), p65 (Cell Signaling Technology 8242S), followed by species-appropriate DyLight™-conjugated anti-IgG (LI-COR Biosciences, Lincoln, NE, USA). Sizes of target proteins were determined by using molecular weight standards (Bio-Rad). Protein band quantitation was carried out with a LI-COR Odyssey CLX imaging system measuring near-infrared fluorescence present in digitized images of membranes and the Image Studio^®^ v4.1 software (LI-COR).

### 4.6. Statistics

Data are expressed as mean ± standard error of the mean (SEM) and analyzed using two-way analysis of variance for multiple comparisons with Bonferroni correction and the Prism 6 (GraphPad Software, La Jolla, CA, USA). The significance for statistical test is shown in the figures, and the differences were deemed significant for probability values (*p*) less than 0.05. The experiments were repeated at least twice, with duplicate or triplicate determinations and consistent results.

## Figures and Tables

**Table 1:** qRTPCR primer sequences.

Gene	Forward (F) Reverse (R)	5′ Primer Sequence 3′
*B2M*	F R	CCGAACATACTGAACTGCTACGTAA CCCGTTCTTCAGCATTTGGA
*βA*	F R	GACGGCCAGGTCATCACTATTG AGGAAGGCTGGAAAAGAGCC
*CCL2*	F R	CACTCACCTGCTGCTACTCA GCTTGGTGACAAAAACTACAGC
*CCL3*	F R	GCCATATGGAGCTGACACCC TAGTCAGGAAAATGACACCTGGC
*CCL5*	F R	TGCCCTCACCATCATCCTCACT GGCGGTTCCTTCGAGTGACA
*FcεRIα*	F R	ATTGTGAGTGCCACCGTTCA GCAGCCAATCTTGCGTTACA
*SphK1*	F R	CGTGGACCTCGAGAGTGAGAA AGGCTTGCTAGGCGAAAGAAG

## Data Availability

Not applicable.
